# Effects of compound organic acid calcium on growth performance, hepatic antioxidation and intestinal barrier of male broilers under heat stress

**DOI:** 10.5713/ajas.19.0274

**Published:** 2019-08-26

**Authors:** Junna He, Lianxiang Ma, Jialing Qiu, Xintao Lu, Chuanchuan Hou, Bing Liu, Dongyou Yu

**Affiliations:** 1 College of Animal Science, Key Laboratory of Animal Nutrition and Feed in East China, Ministry of Agriculture, Zhejiang University, Hangzhou, 310058, China; 2 State Key Laboratory of Food Science and Technology, and Synergetic Innovation Center of Food Safety and Nutrition of Jiangnan University, Wuxi, Jiangsu 214122, China

**Keywords:** Growth Performance, Compound Organic Acid Calcium, Antioxidation, Intestinal Barrier, Heat Stress, Broiler

## Abstract

**Objective:**

The aim of this study was to evaluate the effects of compound organic acid calcium (COAC) on growth performance, hepatic antioxidant status and intestinal barrier of male broilers under high ambient temperature (32.7°C).

**Methods:**

Nine hundred healthy one-d-old Cobb-500 male broiler chicks were randomly assigned into three groups with six replicates of 50 birds each. A basal diet supplemented with 0% (control), 0.4% and 0.8% COAC, respectively were fed to birds for 6 weeks. All treatments were under high ambient indoor temperature of 32.7°C, and had a constant calcium and available phosphorus ratio.

**Results:**

The results showed that, compared with control, the average daily gain of broilers in 0.4% and 0.8% was significantly increased and the ratio of feed to gain in in 0.4% and 0.8% was significantly decreased at 1 to 21, 22 to 42 and 1 to 42 days of age (p<0.05). Compared with control, 0.8% COAC slightly decreased (p = 0.093) the content of malondialdehyde in liver at 42 days of age while 0.4% COAC significantly decreased (p<0.05) the activity of alkaline phosphatase. Furthermore, 0.4% COAC significantly enhanced the intestinal barrier function via increasing jejunal and ileal ocln transcription, promoting jejunal mucin 2 transcription at 42 days of age (p<0.05), and decreasing jejunal toll-like receptor 2 (TLR-2) and ileal TLR-15, inducible nitric oxide synthase compared with control group (p<0.05). Whereas, no significant differences on the transcription of interleukin-1β in jejunum and ileum were observed among three treatments (p>0.05). Overall, heat stress caused by high natural environment temperature may induce the damage to hepatic antioxidation and intestinal barrier.

**Conclusion:**

Dietary inclusion of COAC can improve the tolerance of broilers to thermal environment through the modification of antioxidative parameters in liver and the mRNA expression of genes in intestinal barrier, resulting in an optimal inclusion level of 0.4%.

## INTRODUCTION

Heat stress caused by high ambient temperature and relative humidity is one of the most severe issues in commercial poultry production due to its serious damage on performance, immunity and antioxidant status of broilers [1], gene expression [2], and body temperature at hatching [3], culminating in economic losses. An unreliable housing environment, especially with the additional challenge by the rising temperature due to global warming, could lead to a disruption of the boilers’ body temperature control with dysfunction in the respiratory system and circulation [4]. There are numerous poultry factories in regions across China that suffer from hot summers with at least 30°C for a few hours every day, which have been experiencing technical limitations in attempting make an economic profit. Broilers in these factories would be more vulnerable to heat increments compared to younger birds due to their increased of body mass and development of feather layers as they grow [3]. In order to solve those increasing problems caused by hot climate, different methods have been carried out [5]. Excluding lowering stocking density, dietary supplementation of probiotics, certain acids, organic acids, etc. can be effective methods. As reported, *Bacillus licheniformis* might be useful for ameliorating the adverse influence of heat on the egg production and gut health of laying hens [6], *Lactobacillus* improved the average daily gain (ADG) of broilers [7], methionine hydroxy analogue alleviated major damage of broilers through the action on some genes related to thioredoxin complex activity [8], and organic acid improved microbiological quality of chicken meat [9]. Numerous studies have reported that dietary organic acid or organic acid salts can improve the growth performance, immune status and gut health of broilers [10, 11]. However, few data are available for their effects on broilers under heat stress [12].

Therefore, the main goal of the present study was to investigate the effects of commercial compound organic acid calcium (COAC) on hepatic antioxidant status and intestinal barrier in male broilers under high ambient temperature.

## MATERIALS AND METHODS

### Animals and management

The experiment was conducted in summer. The experimental use of animals and related procedures were performed in accordance with the Chinese Guidelines for Animal Welfare and approved by the Institutional Animal Care and Use Committee of Zhejiang University (Hangzhou, China).

Nine hundred healthy one-d-old Cobb-500 male broiler chicks with an average initial body weight of 45.60±0.58 g were obtained from a commercial hatchery, and randomly distributed into three treatment groups with 6 replicate pens containing 50 birds each. Dietary treatments, with a constant calcium and available phosphorus ratio, were arranged as follows: The control group received the basal diet and the treatment groups received the same basal diet supplemented with 0.4% and 0.8% COAC, respectively. The COAC, provided by Hangzhou Guogu Biological Technology Co., Ltd (Zhejiang, China), is a compound acidifier blend of calcium formate, calcium citrate and calcium lactate with 7:2:1 ratio, and a total calcium content at 26.45%. The experimental diets (starter diets from 1 to 21 d; grower-finisher diets from 22 to 42 d) were formulated to meet the NRC [13] nutrient recommendations for broilers. Ingredients and chemical composition of the diets are presented in Table 1.

Chicks were handled carefully to avoid any pain or injury and placed in a room with adjoining floor pens (50 birds/pen, 0.28 m×0.26 m floor area/bird), which were separated by wire fencing. The room was cleaned and sterilized thoroughly with formaldehyde and potassium permaganate solution (KMnO_4_ + formaldehyde solution) 15 days prior to the arrival of the birds. Birds had *ad libitum* access to diets (in powder form) and water at a constant mean indoor temperature of 32.7°C. The relative humidity was kept from 55% to 65% (Table 2), and the light is nature lighting. Broilers were raised using common management practice for broiler chicks.

### Sample collection and analytical determination

At 21 d and 42 d, after a 12 h feed withdrawn, one bird from each replicate was randomly chosen and killed by cervical dislocation. Then, the liver tissue was collected immediately according to the method of Bai et al [14]. The middle-jejunum and ileum segments were obtained by the method of Adil et al [11]. All samples were quickly snap-frozen in liquid nitrogen and stored at −80°C for further analysis.

### Growth performance

The broilers were weighed at 08:00 am on an empty stomach in terms of replicate at 21 d and 42 d respectively. The amount of feed and feed remaining were recorded for each replicate. The ADG, the average daily feed intake (ADFI), the ratio of feed to gain (F/G) were calculated.

### Liver biochemical and antioxidant indexes

Liver samples were homogenized with ice-cold physiologic saline with the ratio of 1:9, then centrifuged at 2,500 r/min for 15 min under 4°C. The supernatants were collected for the analysis of glutamic oxalacetic transaminase (GOT), aspartate amino transferase (GPT), alkaline phosphatase (AKP), the total antioxidant capacity (T-AOC), total superoxide dismutase (T-SOD), and glutathione peroxidase (GSH-Px) activities, total protein (TP), malondialdehyde (MDA) contents. The kits were provided by Nanjing Jiancheng Biological Engineering Institute (Nanjing, China).

### RNA extraction and quantitative real-time polymerase chain reaction

The mid-jejunum and ileum segments were carefully dissected and rinsed with sterilized saline. Jejunal and ileal mucosa were gently scraped off. All the samples were placed in liquid nitrogen immediately and stored at −80°C till further analysis. Total RNA was extracted using RNAiso Plus method (TaKaRa, Dalian, China). Complementary DNA (cDNA) was synthesized from 1 μg of total RNA using M-MLV reverse transcriptase (TaKaRa, China). Transcriptional changes were then identified by quantitative polymerase chain reaction (PCR), which was performed using the Premix Ex TaqTM with SYBR Green (TaKaRa, China) and the ABI 7500 Fast Real-Time PCR system (Applied Biosystemics, Carlsbad, CA, USA). The thermocycle protocol lasted for 30 s at 95°C, followed by 40 cycles of 5 s denaturation at 95°C, 34 s annealing/extension at 60°C, and then a final melting curve analysis to monitor purity of the PCR product. Primer sequences are presented in Table 3. The 2^–ΔΔCt^ method was used to estimate mRNA abundance. ΔCt is C_t, target_ – C_t, reference_ and ΔΔCt is ΔC_t, treatment_ – ΔC_t,control_. Relative gene expression levels were normalized to those of eukaryotic reference gene β-actin [15].

### Statistical analysis

After being organized by Microsoft Excel 2003 (Microsoft Corp., Redmond, WA, USA), the data were analyzed by one-way analysis of variance of the SPSS 20.0 (SPSS Inc., Chicago, IL, USA) followed by a Duncan’s multiple range test [16]. The results were expressed in terms of the means plus pooled standard error of means. Significance was declared at p<0.05 and statistical tendencies noted at 0.05<p<0.10.

## RESULTS

### Growth performance

Effects of dietary compound organic acid calcium on growth performance of broilers are shown in Table 4. At 1 to 21 d, the ADG in 0.4% COAC and 0.8% COAC were increased by 7.22% and 6.13% (p<0.05) respectively, F/G were decreased by 3.47% and 4.05% (p<0.05) respectively compared with control. At 22 to 42 d, the ADG in 0.4% COAC and 0.8% COAC were improved by 7.84% and 7.86% (p<0.05) respectively, F/G were reduced by 5.24% and 4.76% (p<0.05) respectively, At 1 to 42 d, the ADG in 0.4% COAC and 0.8% COAC were increased by 7.60% and 7.23% (p<0.05) respectively, F/G were decreased by 5.08% and 4.27% (p<0.05) respectively. No remarkable differences (p>0.05) were observed in ADFI at any stage.

### Liver biochemical and antioxidant status

Effects of treatments on liver biochemical and antioxidant status in broilers at d 21 and d 42 under seasonal heat stress are presented in Table 5. No statistical differences were observed in the indicators of GOT, GPT, TP, and AKP (d 21) in liver (p>0.05). However, the activity of AKP was significantly lower in 0.4% COAC compared to 0.8% COAC and control at 42 day of age (p<0.05). Although no significant differences were observed in the activities of T-AOC, T-SOD, GSH-Px, and the content of MDA (d 21) in liver of broilers at two stages (p>0.05), the content of MDA was slightly lower in 0.4% and 0.8% COAC at 42 days of age compared to control group (p = 0.093).

### The expression of genes related to intestinal physical barrier in jejunum of broilers

**Figure 1:**
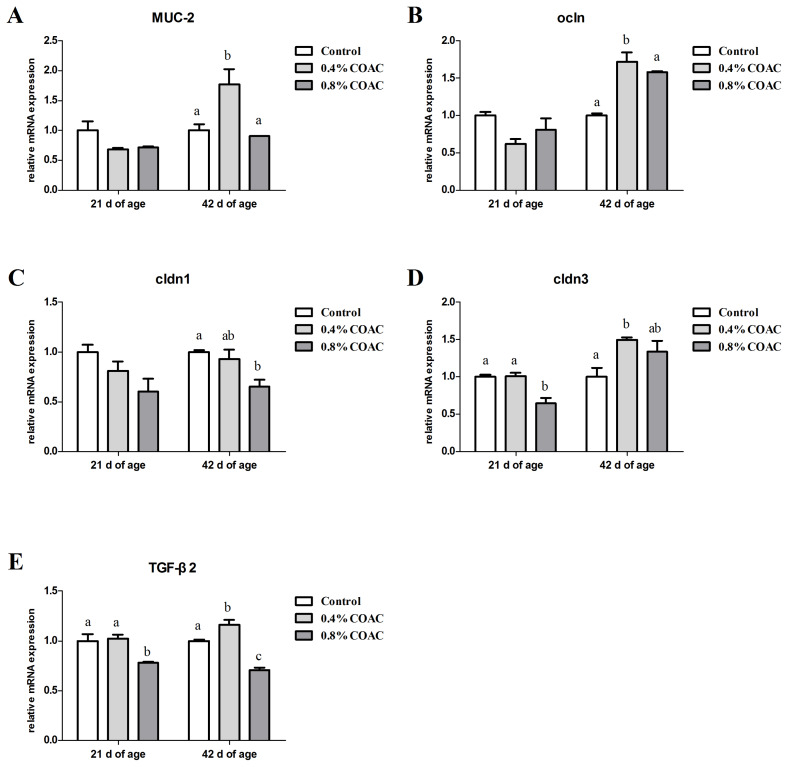
Effects of compound organic acid calcium on MUC-2, ocln, cldn1, cldn3 and TGF-β2 mRNA expression in jejunum of broilers. At 21 and 42 days of age, the expression of MUC-2 (A), ocln (B), cldn1 (C), cldn3 (D) and TGF-β2 (E) were measured by real-time polymerase chain reaction. MUC-2, mucin 2; Ocln, occluding; Cldn, claudin; TGF-β2, transforming growth factor-beta 2. Different letters (a–c) denote a statistical difference (p<0.05).

Changes in the mRNA expression of mucin 2 (*MUC-2*), occludin (*ocln*), claudin 1 (*cldn1*), claudin 3 (*cldn3*) and transforming growth factor-beta β2 (*TGF-β2*) in jejunum of broilers are shown in Figure 1. Compared with control group, at d 21, jejunal cldn3 and *TGF-β2* mRNA expressions of birds fed 0.8% COAC were significantly decreased (p<0.05), while no remarkable differences (p>0.05) were observed on *ocln*, *cldn1* transcriptions among all groups (p>0.05). At d 42, 0.4% COAC significantly increased (p<0.05) the *MUC-2* and *TGF-β2* expressions, whereas, 0.8% COAC reduced (p<0.05) the *cldn1* and *TGF-β2* expressions.

### The expression of genes related to intestinal physical barrier in ileum of broilers

**Figure 2:**
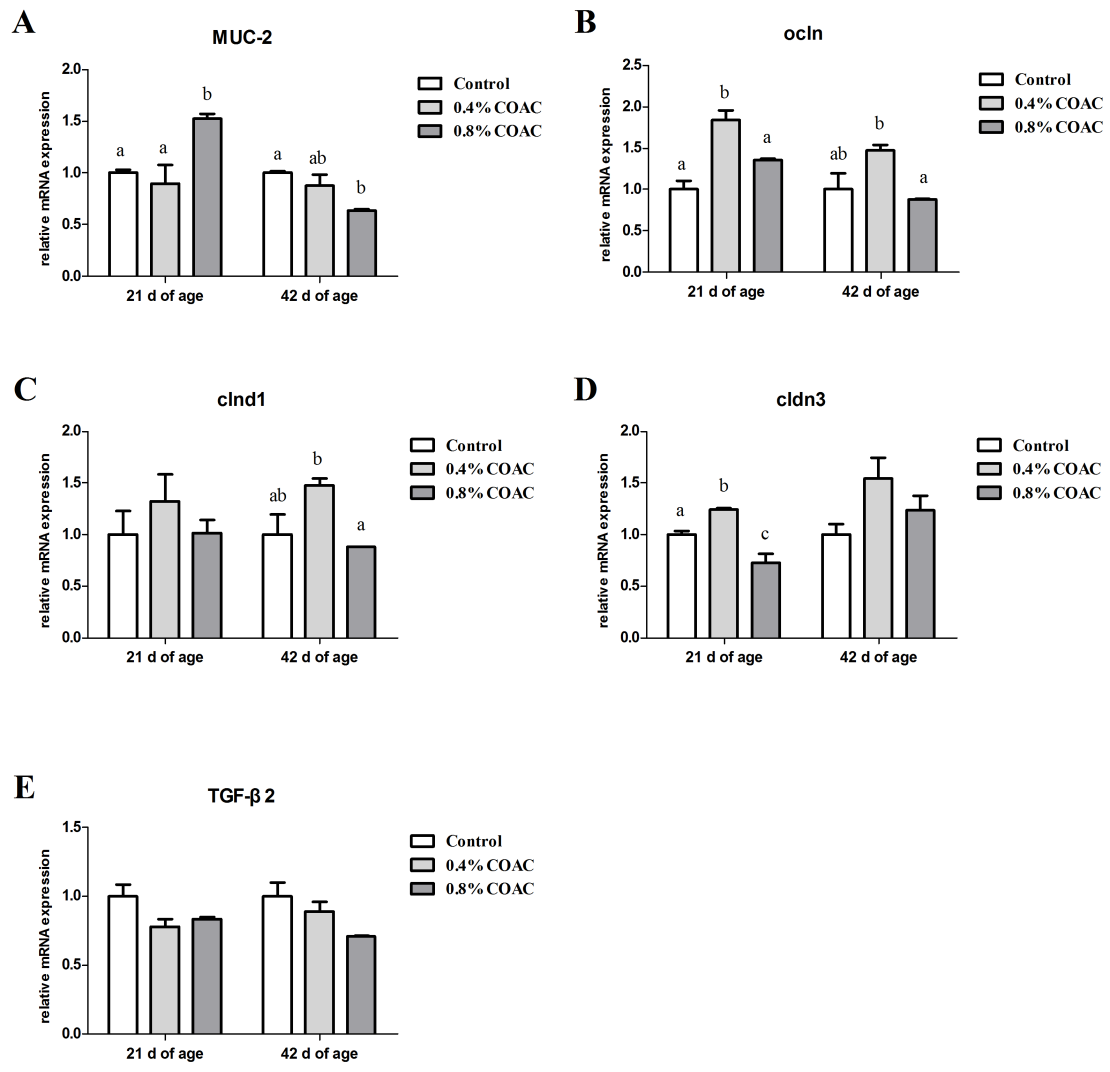
Effects of compound organic acid calcium on ocln, cldn1, cldn3 and TGF-β2 mRNA expression in ileum of broilers. At 21 and 42 days of age, the expression of ocln (A), cldn1 (B), cldn3 (C) and TGF-β2 (D) were measured by real-time polymerase chain reaction. Ocln, occluding; Cldn, claudin; TGF-β2, transforming growth factor-beta 2. Different letters (a–c) denote a statistical difference (p<0.05).

Effect of dietary COAC on the mRNA expressions of *MUC-2*, *ocln*, *cldn1*, *cldn3*, and *TGF-β2* in ileum of broilers are shown in Figure 2. At d 21, the mRNA expressions of *ocln* and *cldn3* in ileum were significantly higher (p<0.05) in birds fed 0.4% COAC than control group. Compared with control and 0.4% COAC, birds in 0.8% COAC had a lower *cldn3* expression and a higher *MUC-2* expression (p<0.05). At d 42, 0.4% COAC obviously elevated (p<0.05) the mRNA expression of *ocln* and *cldn1* compared with 0.8% COAC.

### The expression of genes related to intestinal immunological barrier in jejunum of broilers

**Figure 3:**
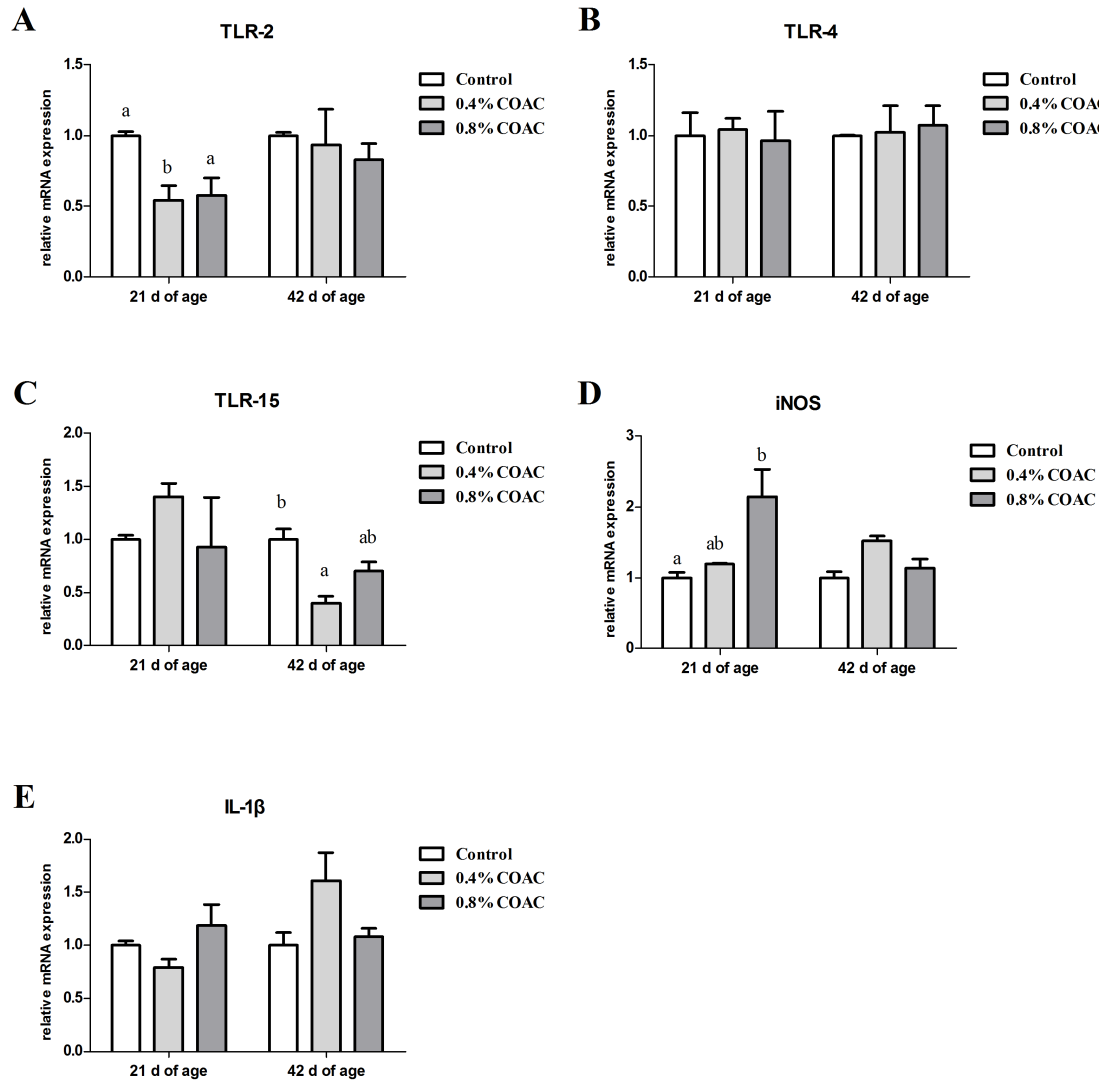
Effects of compound organic acid calcium on TLR, iNOS and IL-1β mRNA expression in jejunum of broilers. At 21 and 42 days of age, the expression of TLR-2 (A), TLR-4 (B), TLR-15 (C), iNOS (D) and IL-1β (E) were measured by real-time polymerase chain reaction. TLR, toll-like receptor; iNOS, inducible nitric oxide synthase; IL-1β, interleukin 1β. Different letters (a, b) denote a statistical difference (p<0.05).

Figure 3 shows the changes in the mRNA expression of toll-like receptor (*TLR*), nitric oxide synthase (*iNOS*) and interleukin-1β (*IL-1β*) in jejunum of broilers. Compared with control group, at d 21, 0.4% COAC significantly decreased (p<0.05) *TLR-2* transcription, and 0.8% COAC notably increased (p<0.05) *iNOS* expression. At d 42, birds fed 0.4% COAC had significantly decreased the mRNA expression of *TLR-15* (p<0.05), while no significant differences were observed in other gene expressions among all treatments (p>0.05).

### The expression of genes related to intestinal immunological barrier in ileum of broilers

**Figure 4:**
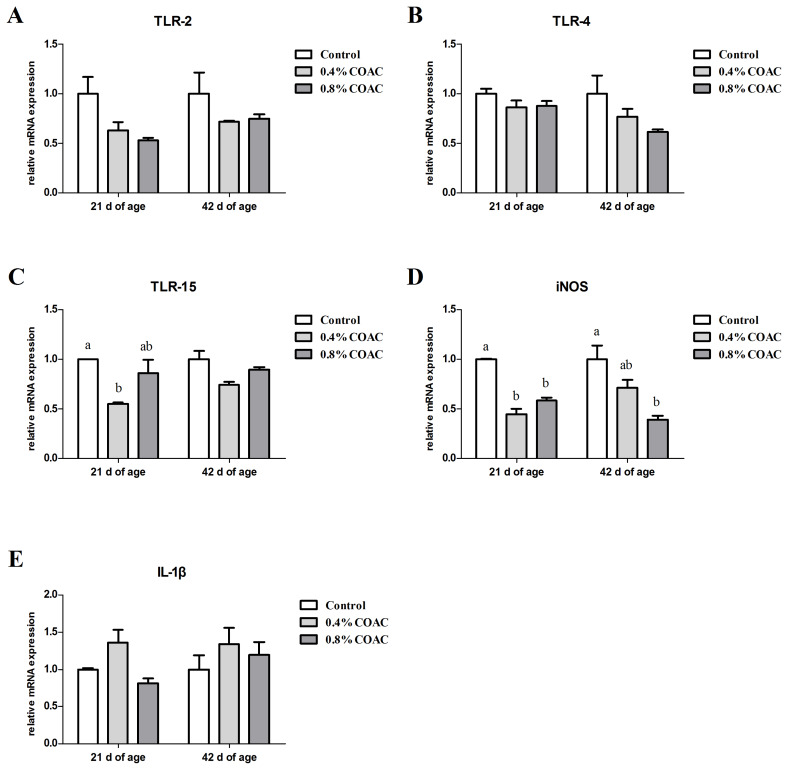
Effects of compound organic acid calcium on TLR, iNOS, and IL-1β mRNA expression in ileum of broilers. At 21 and 42 days of age, the expression of TLR-2 (A), TLR-4 (B), TLR-15 (C), iNOS (D), and IL-1β (E) were measured by real-time polymerase chain reaction. TLR, toll-like receptor; iNOS, inducible nitric oxide synthase; IL, interleukin. Different letters (a, b) denote a statistical difference (p<0.05).

As shown in Figure 4, at d 21, compared with control group, the COAC groups reduced *iNOS* expressions and 0.4% COAC obviously decreased *TLR-15* expression (p<0.05). At d 42, birds fed 0.8% COAC had significantly decreased (p<0.05) mRNA expression of *iNOS*. No significant differences were observed on the mRNA expressions of *TLR-2*, *TLR-4*, and *IL-1β* among all treatments (p>0.05).

## DISCUSSION

The addition of acidifiers or organic acid salts to broiler diets beneficially improves the palatability of the diet while participating in the body's metabolic response and accelerating the body's absorption of nutrients [17]. Thereby promoting broiler weight gain and reducing F/G. Some studies reported that dietary supplemented organic acids can significantly increase daily gain and feed conversion in broilers [10], which is agreement with our results. In the present study, there was no significant difference in ADFI observed between experiment groups, which is consistent with studies reported by Sultan et al [18] and Gunal et al [19]. While, the studies by Chowdhury et al [10] and Haque et al [20] showed that the addition of organic acids to the diet increased the feed intake of broilers. And other studies found that dietary supplement organic acids reduced the feed intake of broilers [17,21], which might be related to the type of organic acid, the amount of addition, the feeding environment and the feeding method [18,20,22].

The structure and physiology of the cells can be affected by high temperature, causing impairment of transcription, oxidative metabolism, membrane structure and function [23] and gene expression [2]. Also, heat stress can result in oxidative stress and induce cells to generate small amounts of free radicals or reactive oxygen species (ROS) [5], which may damage biological macro-molecules and tissues if not controlled [24]. However, these damages might be minimized via antioxidant defense systems, including antioxidant enzymes such as SOD, GSP-Px, etc. [25]. The biochemical and antioxidant indices play a vital role in protecting cellular and tissue damage from harmful effects of ROS [25], and are usually used as indicators of the health condition of animals [5]. The activities of GOT and GPT can reflect the functions and integrity of tissues like liver [26]. AKP performs lipid transportation in the intestine, calcification in bone and is present in liver, and usually remains normal or moderately increased in acute viral hepatitis [27], decreased in hypothyroidism and pernicious anaemia [28]. In the present study, COAC did not influence the activities of GOT, GPT, TP, and AKP in liver at d 21. While the AKP activity was significantly decreased in 0.4% COAC, which may be have a protective effect in the liver. MDA, one of the important metabolites of lipid peroxidation, can serve as an indicator of tissue injury. The antioxidant enzymes like T-AOC, T-SOD, and GSH-Px can minimize the oxidative damage by protecting the cell against cellular oxidants and preventing the accumulation of oxidatively injured molecules [29]. Although no significant differences were observed on the activities of T-AOC, T-SOD, and GSH-Px along with the content of MDA (d 21) in liver of broilers, the content of MDA was slightly lower in both 0.4% and 0.8% COAC compared with control group, which is in agreement with the results of Seven et al [29] and Ma et al [30] where heat stress increased the concentration of MDA in liver of both broilers and laying hens.

The integrity of the seal of the paracellular pathway between cells is crucial for the epithelial cell layer to function properly [15], and this seal is achieved by a physical barrier, especially tight junctions [31]. The functional barrier of the intestinal epithelial separates the intestine from the outside world, and requires the formation of tight junctions that allow cells to adhere tightly to each other and control the intestinal permeability [32]. Tight junctions such as ocln, and cldn are structural and functional proteins [15], among which the most abundant mucin is *MUC-2*, that can create the first line of defense against microbial encroachment [33]. In the present study, the transcript levels of all the tested tight junctions were up-regulated by COAC and the effect was more obvious when birds were fed 0.4% COAC. A damaged intestinal epithelial integrity may facilitate the invasion of endotoxins from gut microbes, leading to a local imbalance of anti- and pro-inflammatory molecules in the intestines [34]. COAC improved the intestinal physical barrier and may also participate in enhancing the intestinal immunological barrier function.

In addition, TLRs are a group of evolutionarily conserved membrane receptors broadly expressed on various innate immunity cells and non-immune cells, they function as primary sensors that can initiate innate immune responses via responding to pathogen-associated molecular patterns from bacteria, viruses, etc. [35]. Moreover, these *TLRs* such as *TLR2* and *TLR4* are expressed in pro-inflammatory macrophages and vascular, which would release more *TLRs* when tissues were injured [36], as *iNOS* and *IL-1β*. In the current study, we found that COAC significantly decreased the expressions of *TLR-2*, *TLR-15* and pro-inflammatory cytokine *iNOS*, which might be related to the stimulation of organic acid calcium on immune related organs [37].

## CONCLUSION

Dietary supplementation of compound organic acid calcium can improve the growth performance and alleviate the damages in broilers under heat stress. The effect was demonstrated by increasing the immunological competence, regulating *TLR-2*, *TLR-4*, and *MUC-2* expressions, resulting in the improvement of broilers tolerance to the damages in liver and small intestine. However, only limited studies on the molecular mechanism of dietary organic acid or organic acid salts against the damage of heat stress in broilers have been carried out, which needs further exploration.

## Figures and Tables

**Table 1:** Ingredients and nutrient levels of the experimental diets (%, as fed basis)

Item	Starter phase1) (1 to 21 d)			Grower-finisher phase1) (22 to 42 d)		
	
	Control	0.4% COAC	0.8% COAC	Control	0.4% COAC	0.8% COAC
Ingredients						
Corn	60.00	60.00	60.00	66.00	66.00	66.00
Soybean meal	28.50	28.50	28.50	24.00	24.00	24.00
Fish meal	2.00	2.00	2.00	1.00	1.00	1.00
Wheat middlings	4.50	4.50	4.50	4.00	4.00	4.00
Salt	0.30	0.30	0.30	0.30	0.30	0.30
Choline chloride (50%)	0.15	0.15	0.15	0.10	0.10	0.10
Calcium hydrophosphate	1.20	1.20	1.20	1.00	1.00	1.00
Limestone	1.20	0.90	0.60	1.20	0.90	0.60
Compound organic acid calcium	0.00	0.40	0.80	0.00	0.40	0.80
Zeolite power	1.15	1.05	0.95	1.40	1.30	1.20
Premix2)	1.00	1.00	1.00	1.00	1.00	1.00
Total	100.00	100.00	100.00	100.00	100.00	100.00
Nutrient levels3)						
ME (MJ/kg)	11.73	11.73	11.73	11.91	11.91	11.91
Crude protein	18.86	18.86	18.86	16.69	16.69	16.69
Lysine	1.21	1.21	1.21	1.08	1.08	1.08
Methionine	0.57	0.57	0.57	0.45	0.45	0.45
Calcium	0.98	1.00	0.98	0.87	0.87	0.86
Total phosphorus	0.70	0.70	0.70	0.61	0.61	0.61
Aailable phosphorus	0.46	0.46	0.46	0.39	0.39	0.39

COAC, compound organic acid calcium; ME, metabolizable energy.

1) Control = basal diet without any feed additive; 0.4% COAC = basal diet + 0.4% compound organic acid calcium; 0.8% COAC = basal diet + 0.8% compound organic acid calcium.

2) Premix supplied the following per kilogram of diet: vitamin A 25,000 IU; vitamin D 5,000 IU; vitamin E (DL-α-tocopheryl acetate), 12.5 IU; vitamin K (menadione) 1.25 mg; vitamin B_1_ (thiamine) 1.0 mg; vitamin B_2_ (riboflavin) 8 mg; vitamin B_6_ (pyridoxine) 3.0 mg; vitamin B_12_ (cyanocobalamin) 15 μg; folic acid 250 μg; nicotinic acid 17.5 mg; calcium pantothenate 12.5 mg; Fe 80 mg; Cu 10 mg; Mn 80 mg; Zn 80 mg; Se 0.15 mg; I 0.35 mg; phytase 500 U.

3) Calcium was measured value, while the others were calculated values.

**Table 2:** Mean outdoor and indoor temperature and relative humidity

Items	Mean outdoor temperature (°C)	Mean indoor temperature (°C)	Relative humidity (%)
The 1st wk	29.86	30.71	55.71
The 2nd wk	33.21	34.00	58.43
The 3rd wk	37.00	38.00	64.00
The 4th wk	32.29	33.00	62.57
The 5th wk	30.50	31.43	62.86
The 6th wk	27.79	29.00	60.86
Mean	31.77	32.70	60.74

**Table 3:** Gene names and primer sequences

Gene	Primer sequence 5'-3'
*MUC-2*	F: GCCTGCCCAGGAAATCAAG
	R: CGACAAGTTTGCTGGCACAT
*Ocln*	F: GAGCCCAGACTACCAAAGCAA
	R: GCTTGATGTGGAAGAGCTTGTTG
*Cldn1*	F: TGGCCACGTCATGGTATGG
	R: AACGGGTGTGAAAGGGTCATAG
*Cldn3*	F: AATGCGCCATCTCTGCAAAC
	R: GTTTCTCCGCCAGACTCTCC
*TLR2*	F: TGTTCCTGTTCATCCTCATCCT
	R: AGTTGGAGTCGTTCTCACTGT
*β-actin*	F: TATGTGCAAGGCCGGTTTC
	R: TGTCTTTCTGGCCCATACCAA
*TLR4*	F: GAATGACACGGACACTCTT
	R: ACATAGGAACCTCTGACAAC
*TLR15*	F: CTTGTCGTTCTGGTGCTAA
	R: ATCGTGCTCGCTGTATGA
*IL-1β*	F: CGACATCAACCAGAAGTGCTT
	R: GTCCAGGCGGTAGAAGATGA
*iNOS*	F: TACTCTTGGCGTCATTACTC
	R: GCATAGATCACAGTCACCTT
*TGF-β2*	F: TCTCGGAGCAGCGGATAGA
	R: AATCCAAGGTTCCTGTCTCTGT

F, forward; R, reverse; *MUC-2*, mucin 2; *Ocln*, occluding; *Cldn*, claudin; *TLR*, toll-like receptor; *IL*, interleukin; *iNOS*, inducible nitric oxide synthase; *TGF-β2*, transforming growth factor-beta 2.

**Table 4:** Effects of dietary compound organic acid calcium on growth performance of broilers

Items	21 days of age1)			SEM	p-value

	Control	0.4% COAC	0.8% COAC		
Body weight (g)					
1 day of age	45.80	45.19	45.40	0.35	0.52
21 days of age	563.45^a^	600.15^b^	594.71^b^	9.91	0.04
42 days of age	1,498.89^a^	1,608.86^b^	1,603.45^b^	35.31	0.01
1 to 21 days of age					
ADFI (g)	42.68	44.02	43.43	0.74	0.50
ADG (g)	24.65^a^	26.43^b^	26.16^b^	0.48	0.04
F/G	1.73^b^	1.67^a^	1.66^b^	0.01	0.01
22 to 42 days of age					
ADFI (g)	93.43	95.59	95.98	1.62	0.55
ADG (g)	44.54^a^	48.03^b^	48.04^b^	1.51	0.02
F/G	2.10^b^	1.99^a^	2.00^a^	0.04	0.01
1 to 42 days of age					
ADFI (g)	67.99	69.77	69.71	0.95	0.37
ADG (g)	34.60^a^	37.23^b^	37.10^b^	0.85	0.01
F/G	1.97^b^	1.87^a^	1.88^a^	0.03	0.02

Values reported as means (n = 6).

COAC, compound organic acid calcium; SEM, standard error of means for 6 broilers each; ADFI, the average daily feed intake; ADG, the average daily gain; F/G, the ratio of feed gain.

1) Control = basal diet without any feed additive; 0.4% COAC = basal diet + 0.4% compound organic acid calcium; 0.8% COAC = basal diet + 0.8% compound organic acid calcium.

a,b Means in the same row with different superscripts differ statistically (p<0.05).

**Table 5:** Effects of dietary compound organic acid calcium supplementation on liver biochemical and antioxidant status in broilers under seasonal heat stress

Items	21 days of age1)			SEM	p-value	42 days of age1)			SEM	p-value
	
	Control	0.4% COAC	0.8% COAC			Control	0.4% COAC	0.8% COAC		
Biochemical constituents										
GOT (U/L)	29.87	30.48	35.28	2.648	0.302	20.55	19.95	20.5	0.833	0.867
GPT (U/L)	2.2	1.54	1.26	0.35	0.141	2.43	1.70	2.46	0.473	0.461
AKP (U/L)	17.07	19.63	17.06	2.106	0.672	87.32^b^	65.93^a^	94.29^b^	8.007	0.022
TP (g/L)	11.12	11.5	11.69	1.27	0.700	10.81	10.86	19.96	1.260	0.970
Antioxidants enzymes										
T-AOC (U/mL)	1.39	1.53	1.46	0.062	0.282	2.34	2.96	2.53	0.419	0.626
T-SOD (U/mL)	295.09	324.75	323.76	20.788	0.584	95.06	99.43	104.08	7.534	0.755
GSH-Px (U/L)	23.02	23.06	23.15	1.453	0.998	25.97	24.03	29.9	3.194	0.476
MDA (nmol/mL)	0.57	0.38	0.45	0.071	0.143	2.02	1.62	1.31	0.236	0.093

Values reported as means (n = 6).

COAC, compound organic acid calcium; SEM, standard error of means for 6 broilers each; GOT, glutamic oxalacetic transaminase; GPT, aspartate amino transferase; AKP, alkaline phosphatase; TP, total protein; T-AOC, total antioxidant capacity; T-SOD, total superoxide dismutase; GSH-Px, glutathione peroxidase; MDA, malondialdehyde.

1) Control = basal diet without any feed additive; 0.4% COAC = basal diet + 0.4% compound organic acid calcium; 0.8% COAC = basal diet + 0.8% compound organic acid calcium.

a,b Means in the same row with different superscripts differ statistically (p<0.05).
